# Autophagosome Precursor Maturation Requires Homotypic Fusion

**DOI:** 10.1016/j.cell.2011.06.023

**Published:** 2011-07-22

**Authors:** Kevin Moreau, Brinda Ravikumar, Maurizio Renna, Claudia Puri, David C. Rubinsztein

**Affiliations:** 1 Department of Medical Genetics, Cambridge Institute for Medical Research, Wellcome/MRC Building, Addenbrooke's Hospital, Hills Road, Cambridge CB2 20Y, UK

## Abstract

Autophagy is a catabolic process in which lysosomes degrade intracytoplasmic contents transported in double-membraned autophagosomes. Autophagosomes are formed by the elongation and fusion of phagophores, which can be derived from preautophagosomal structures coming from the plasma membrane and other sites like the endoplasmic reticulum and mitochondria. The mechanisms by which preautophagosomal structures elongate their membranes and mature toward fully formed autophagosomes still remain unknown. Here, we show that the maturation of the early Atg16L1 precursors requires homotypic fusion, which is essential for subsequent autophagosome formation. Atg16L1 precursor homotypic fusion depends on the SNARE protein VAMP7 together with partner SNAREs. Atg16L1 precursor homotypic fusion is a critical event in the early phases of autophagy that couples membrane acquisition and autophagosome biogenesis, as this step regulates the size of the vesicles, which in turn appears to influence their subsequent maturation into LC3-positive autophagosomes.

## Graphical abstract

**Graphical Abstract:**

**Figure:**
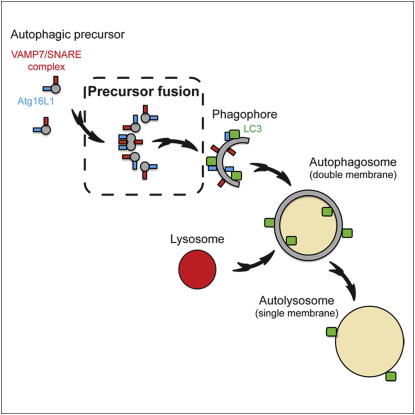

**Highlights:**

► Homotypic fusion of Atg16L1 vesicles enables their maturation into autophagosomes ► VAMP7 regulates Atg16L1 vesicle homotypic fusion and autophagosome formation ► Plasma membrane is a likely source of SNAREs required for Atg16L1 vesicle fusion ► Starvation, which induces autophagy, triggers Atg16L1 vesicle homotypic fusion

## Introduction

Macroautophagy, which we will refer to as autophagy, is a catabolic process in which cytoplasmic materials are engulfed by double membrane structures, which form autophagosomes. Autophagosomes ultimately fuse with lysosomes to form autolysosomes, where their contents are degraded. Autophagy regulates numerous physiological and pathological processes, including survival during nutrient starvation, elimination of intracytoplasmic aggregate-prone proteins and pathogens, and innate and acquired immunity (Levine and Deretic, 2007; Mizushima et al., 2008; Ravikumar et al., 2009).

Autophagosome formation occurs through dynamic rearrangements of cellular membrane structures, initiated by the emergence of an isolation membrane called a phagophore. The phagophore membranes extend in a manner that is not understood and fuse to create a double-membrane autophagosome (Mizushima et al., 2008; Ravikumar et al., 2009; Xie and Klionsky, 2007). More than 30 autophagy-related proteins (Atg) have been discovered to regulate autophagy. Some of these proteins contribute to two conjugation reactions that are required for autophagosome formation. The first involves Atg12 conjugation to Atg5 (Xie and Klionsky, 2007). This Atg12-Atg5 conjugate interacts noncovalently with Atg16L1 to form a complex essential for the phagophore expansion. The complexes formed by Atg16L1-Atg12-Atg5 are localized to the phagophore and dissociate upon completion of autophagosome formation. Thus, vesicular structures that contain these proteins are considered to be phagophores (and not completed autophagosomes). A second conjugation reaction involving Atg8/LC3 contributes to the completion of autophagosome formation. LC3 is cleaved by Atg4 to form cytosolic LC3-I, which is covalently conjugated to the phosphatidylethanolamine to form membrane-associated LC3-II (Xie and Klionsky, 2007). LC3-II is the only known protein that specifically associates with autophagosomes. Thus, LC3-II levels correlate with autophagic vesicle numbers, which can be assessed by western blotting or by scoring LC3-positive vesicle numbers (Rubinsztein et al., 2009).

Autophagosome membranes have been proposed to originate from a number of sources, including the endoplasmic reticulum (ER), Golgi, mitochondria, and the plasma membrane (Hailey et al., 2010; Hayashi-Nishino et al., 2009; Ravikumar et al., 2010; van der Vaart et al., 2010; Yen et al., 2010; Yla-Anttila et al., 2009). Clathrin-mediated endocytosis provides membrane that contributes to the formation of Atg16L1-positive, LC3-negative preautophagosomal structures (which are also negative for EEA-1 and LAMP1) and the subsequent generation of phagophores (Atg16L1-positive, LC3-positive) and mature autophagosomes (Atg16L1-negative, LC3-positive) (Ravikumar et al., 2010). The processes mediating the maturation of preautophagosomal structures toward phagophores and mature autophagosomes are unknown and represent an important gap in the understanding of autophagosome biogenesis.

In this study, we reveal that autophagic activity requires homotypic fusion of Atg16L1 precursors in order to form mature autophagosomes. This step is dependent on the SNARE protein VAMP7 together with partner SNAREs. The homotypic fusion and velocity of Atg16L1 precursors increase when autophagy is stimulated by starvation or rapamycin treatment.

## Results

### VAMP7 Colocalizes with Atg16L1 and Atg5 Proteins

**Figure 1:**
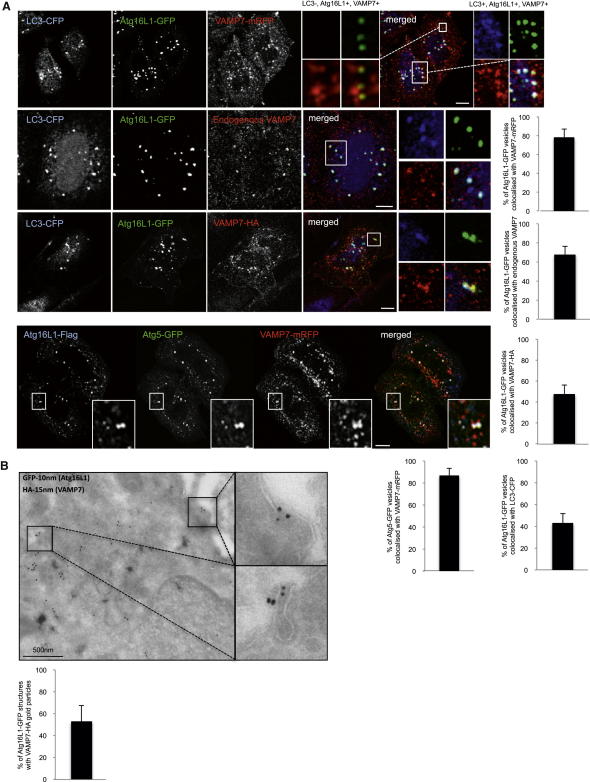
VAMP7 Colocalizes with Atg16L1 and Atg5 (A) Colocalization between LC3-CFP, Atg16L1-GFP, Atg16L1-Flag, Atg5-GFP, VAMP7-mRFP, endogenous VAMP7, and stably expressed VAMP7-HA. Cells were transfected for 20 hr. Confocal colocalization is indicated. At least 20 cells were analyzed in each condition. Error bars = standard deviation (SD). Scale bar, 5 μm. (B) HeLa cells stably expressing VAMP7-HA were transfected for 20 hr with Atg16L1-GFP. The cells were processed for double immunogold labeling with anti-HA antibodies (15 nm gold particles) and anti-GFP antibodies (10 nm gold particles) for electron microscopy. Colocalization between Atg16L1 structures with VAMP7-HA gold particles is shown. n = 17 cells. Scale bar, 500 nm. Error bars = standard error of the mean (SEM). See also Figure S1.

**Figure S1:**
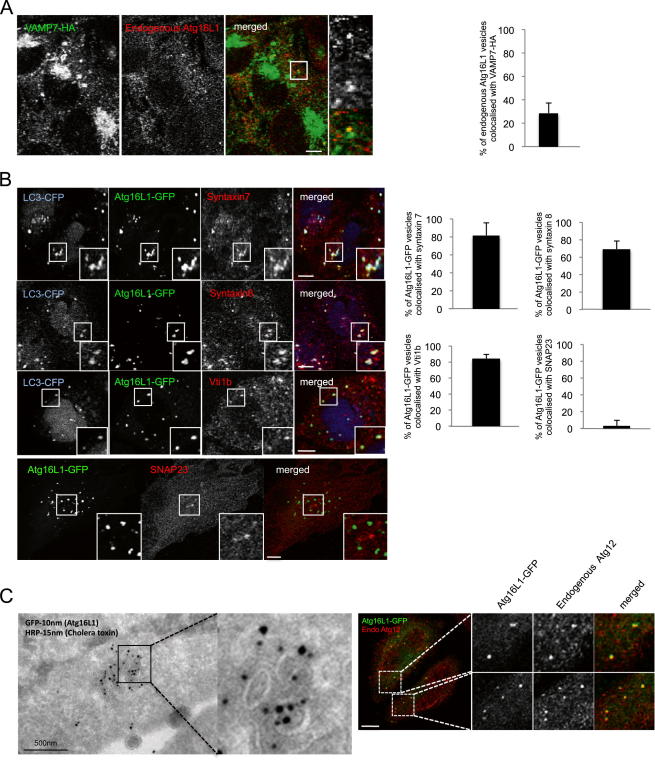
Colocalization Analysis between VAMP7, Syntaxin 7, Syntaxin 8, Vti1b, SNAP23, and Atg16L1, Related to Figure 1 (A) Colocalization between endogenous Atg16L1 and stably expressed VAMP7-HA. HeLa cells stably expressing VAMP7-HA were fixed and subjected to confocal analysis. Colocalization efficiency between endogenous Atg16L1 and VAMP7-HA is shown. n = 20 cells. Error bars throughout this figure are SD. Scale bar, 5 μm. (B) Colocalization between LC3-CFP, Atg16L1-GFP and endogenous syntaxin 7 (Stx7), syntaxin 8 (Stx8), Vti1b and SNAP23. HeLa cells were transiently transfected for 20 hr with LC3-CFP and Atg16L1-GFP. Cells were fixed and subjected to confocal analysis after an immunostaining of Stx7, Stx8, Vti1b, or SNAP23. Colocalization efficiency is shown. n ≥ 20 cells in each condition. Scale bars, 5 μm. (C) HeLa cells transfected for 20 hr with Atg16L1-GFP were processed for double immunogold labeling with anti-HRP antibodies (15 nm gold particles; cholera toxin, used as an endocytic tracer) and anti-GFP antibodies (10 nm gold particles) for electron microscopy or were processed for immunofluorescence analysis after an immunostaining for endogenous Atg12. Colocalization between Atg16L1-GFP and the cholera toxin on vesicular structures is shown on the electronic microscopy picture. Colocalization between Atg16L1-GFP and endogenous Atg12 is shown on confocal pictures. Scale bar, 5 μm.

To understand how Atg16L1 precursors (including small vesicles derived from the plasma membrane) mature toward phagophores and subsequently to a fully formed autophagosome, we hypothesized that fusion events may occur between early precursors. Vesicle fusion depends on multiple classes of proteins, including SNARE proteins, which play a major role by docking vesicles with their target compartments and by catalyzing the fusion of the opposing membranes. As part of an ongoing screen, we identified the SNARE VAMP7 (Tetanus Neurotoxin-Insensitive Vesicle-Associated Membrane Protein) as a candidate regulator of autophagosome formation. To study the potential role of VAMP7 at the Atg16L1 precursor and/or phagophore stage, we performed colocalization analysis using endogenous or exogenous VAMP7, Atg16L1, and LC3 (Figure 1A and Figure S1A available online). Atg16L1-GFP colocalized with VAMP7-mRFP in LC3-negative structures (top panels) (Figure 1A), but also with vesicles containing both VAMP7-mRFP and LC3-CFP (Figure 1A), indicating the localization of VAMP7 at the Atg16L1 precursor and phagophore levels. Atg16L1-GFP colocalized in a similar fashion with endogenous VAMP7 or stably expressed VAMP7-HA (Figure 1A). Atg16L1-GFP vesicles containing VAMP7 also colocalized with Atg5-GFP, another marker of autophagic precursors (Figure 1A). Endogenous Atg16L1 colocalized with stably expressed VAMP7-HA (Figure S1A). VAMP7 is associated with different SNARE partners to form an organelle-specific fusion complex, which ensures that vesicles fuse only with appropriate acceptor membranes (Hong, 2005). We observed colocalization between Atg16L1-GFP and endogenous syntaxin 7, syntaxin 8, and Vti1b, partner SNAREs of VAMP7 localized in the endosomal compartment (Figure S1B) (Bonifacino and Glick, 2004). In contrast, Atg16L1-GFP did not colocalize with SNAP23, a partner SNARE of VAMP7 localized at the plasma membrane involved in exocytosis (Figure S1B) (Bonifacino and Glick, 2004; Guo et al., 1998; Hong, 2005). We confirmed the localization of VAMP7 on Atg16L1 precursors by immunoelectron microscopy in VAMP7-HA stable cells exogenously expressing Atg16L1-GFP (Figure 1B). Thus, VAMP7 and its partner SNAREs syntaxin 7, syntaxin 8, and Vti1b are localized on Atg16L1 precursors and on phagophores.

We confirmed our recent data that Atg16L1-GFP dots obtained after overexpression are true autophagic structures, as they colocalize with endogenous Atg12 using confocal analysis and decorate vesicles visualized by immunoelectron microscopy (Figure S1C) (Ravikumar et al., 2010).

### VAMP7 Regulates Autophagic Activity

**Figure 2:**
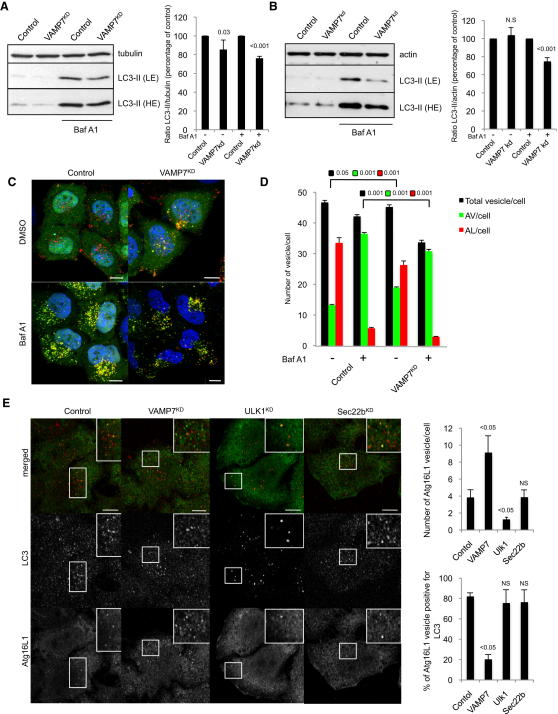
VAMP7 Regulates Autophagic Activity (A) HeLa cells transfected with control or VAMP7 siRNA for 5 days were treated during the last 16 hr with bafilomycin A1 (Baf A1) or DMSO. Means ± SD of the percentage of LC3-II/tubulin ratio from three independent experiments are shown. LE (light exposure of film), HE (longer exposure of film). (B) HeLa cells transfected with control or VAMP7 siRNA for 5 days were starved and treated during the last 16 hr with bafilomycin A1 (Baf A1) or DMSO. Means ± SD of LC3-II/actin ratio from three independent experiments. (C) HeLa cells stably expressing LC3-GFP-mRFP were transfected with control or VAMP7 siRNA for 5 days and treated during the last 16 hr with bafilomycin A1 (Baf A1) or DMSO. Cells were fixed and subjected to automated counting of LC3 vesicles. Representative confocal pictures are shown. Scale bar, 5 μm. (D) Quantification of total number of vesicles/cell, autophagosomes/cell (AV/cell; LC3 GFP^+^/mRFP^+^), and autolysosomes/cell (AL/cell; LC3 GFP^−^/mRFP^+^) from (C) is shown. At least 2000 cells were counted per experiment; values are mean ± SEM of one representative experiment of three independent experiments performed. (E) HeLa cells transfected with control, VAMP7, Ulk1, or Sec22B siRNA for 5 days were immunolabeled for endogenous LC3 (red) and Atg16L1 (green). Representative confocal pictures and quantification of Atg16L1 vesicles are shown. At least 50 cells were counted per experiment; values are mean ± SD of the number of Atg16L1 vesicles per cell obtained from two independent experiments. Colocalization efficiency between Atg16L1 and LC3 is also shown. At least 50 cells were counted per experiment and the data represent the percentage of Atg16L1 vesicles containing LC3 ± SD obtained from two independent experiments. Scale bar, 5 μm. NS—not significant. See also Figure S2.

**Figure S2:**
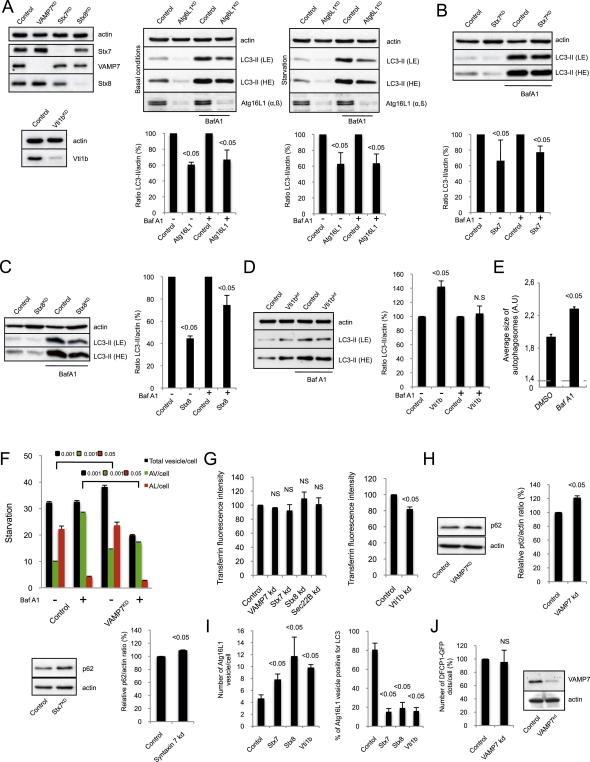
VAMP7, Syntaxin 7, Syntaxin 8, and Vti1b Regulate Autophagic Activity, Related to Figure 2 (A) HeLa cells transfected with two rounds of control, VAMP7, syntaxin 7 (Stx7), syntaxin 8 (Stx8), Vti1b, or Atg16L1 siRNA for 5 days were lysed and subjected to western blotting with the indicated antibodies. For Atg16L1 knockdown, the cells were starved when indicated using HBBS medium treatment for 8 hr and treated during the last 4 hr with bafilomycin A1 (Baf A1) or DMSO (vehicle). Quantification of LC3-II/actin ratio is shown for Atg16L1 knockdown. The data represent the mean ± SD of the percentage of LC3-II/actin ratio obtained from three independent experiments. (B–D) HeLa cells transfected with two rounds of control, Stx7, Stx8, or Vti1b siRNA for 5 days were treated during the last 16 hr with bafilomycin A1 (Baf A1) or DMSO (vehicle). Cells were lysed and subjected to western blotting with the indicated antibodies. Quantification of LC3-II/actin ratio is shown. The data represent the mean ± SD of the percentage of LC3-II/actin ratio obtained from three independent experiments. (E) HeLa cells stably expressing LC3-GFP-mRFP were treated as indicated during the last 16 hr with bafilomycin A1 (Baf A1), fixed and subjected to a Cellomics ArrayScan VTI HCS Reader analysis. Quantification of the size of autophagic vesicles is shown. The data represent the mean ± SEM of the size of autophagic vesicles (A.U; arbitrary unit) of 1 experiment representative of three independent experiments performed where minimums of 2000 cells were automatically analyzed. (F) HeLa cells stably expressing LC3-GFP-mRFP were transfected with two rounds of control or VAMP7 siRNA for 5 days and treated during the last 16 hr with bafilomycin A1 (Baf A1) or DMSO. Cells were starved for 4 hr, fixed, and subjected to automatic counting of LC3 vesicles. Quantification of total number of vesicles/cell, autophagosomes/cell (AV/cell) and autolysosomes/cell (AL/cell) is shown. At least 2000 cells were counted per experiment and the values are mean ± SEM of one representative experiment of three independent experiments performed. (G) HeLa cells transfected with two rounds of control, VAMP7, Stx7, Stx8, Sec22B, or Vti1b siRNA for 5 days were subjected to Alexa 488-labeled transferrin internalisation assay and the amount of fluorescent-transferrin internalised under different knockdown conditions was measured by FACS and presented in the graph. NS—not significant. (H) HeLa cells transfected with two rounds of control, VAMP7 or Stx7 siRNA for 5 days were lysed and subjected to western blotting with the indicated antibodies. Quantification of p62/actin ratio is shown. The data represent the mean ± SD of the percentage of p62/actin ratio obtained from three independent experiments. (I) HeLa cells transfected with two rounds of control, Stx7, Stx8, or Vti1b siRNA for 5 days were fixed and subjected to immunolabeling for endogenous LC3 and Atg16L1. At least 50 cells were counted per experiment and the values are mean ± SD of the number of Atg16L1 vesicles per cell obtained from two independent experiments. The colocalization efficiency between Atg16L1 and LC3 is also shown. At least 50 cells were counted per experiment and the data represent the percentage of Atg16L1 vesicles containing LC3 obtained from two independent experiments. Scale bar, 5 μm. (J) DFCP1-GFP cells transfected with two rounds of control or VAMP7 siRNA for 5 days were fixed and subjected to confocal analysis for DFCP1 dots counting. At least 50 cells were counted per experiment and the data are mean ± SD of the number of DFCP1 dots per cell (in percentage, relative to control) obtained from two independent experiments.

Having shown that VAMP7, syntaxin 7, syntaxin 8, and Vti1b localized to vesicles involved in the early steps of autophagosome biogenesis, we next studied the roles of these SNAREs in autophagic activity. We first tested the effect of VAMP7, syntaxin 7, syntaxin 8, and Vti1b knockdown (Figure S2A) on autophagosome formation by studying LC3-II accumulation on western blot. LC3-II changes can be the consequence of autophagosome formation and/or degradation. To discriminate between these possibilities, LC3-II levels can be assessed in the presence of saturating concentrations of bafilomycin A1 (Baf A1), a potent inhibitor of the vacuolar H^+^ ATPase that inhibits the degradation of LC3-II (Rubinsztein et al., 2009). Knockdown of VAMP7, syntaxin 7, and syntaxin 8 decreased LC3-II levels in the presence or absence of Baf A1, indicating an inhibition of autophagosome formation (Figure 2A, Figures S2B and S2C). The magnitude of this inhibition was not dissimilar to that seen with knockdown of Atg16L1 (a key autophagy protein) (Xie and Klionsky, 2007) (Figure S2A). The effects of VAMP7 knockdown on autophagosome formation (LC3-II levels under Baf A1 conditions) were also obvious under conditions of autophagy induction by starvation, similar to that seen with knockdown of Atg16L1 (Figure 2B and Figure S2A), in which the knockdown decreased new autophagosome formation by about 30%. Vti1b knockdown increased LC3-II levels in the absence of Baf A1, as described previously, because it regulates autophagosome-lysosome fusion (Figure S2D) (Furuta et al., 2010). Vti1b knockdown did not cause any obvious decrease in LC3-II levels in the presence of Baf A1 (Figure S2D). This may be because this perturbation does not affect LC3-II formation. However, it may be difficult to exclude the possibility that Vti1b knockdown may affect both autophagosome formation and autophagosome-lysosome fusion (autophagosome degradation), given that the extent of knockdown that impacts on the formation versus degradation steps may differ (as we have seen with clathrin, for example; Ravikumar et al., 2010), and for certain SNAREs, some function can be preserved in cells even when there are only a few molecules left (Bethani et al., 2009). Furthermore, even a 16 hr treatment with Baf A1 may not be sufficient to overcome the effects of a 5 day siRNA knockdown protocol for an autophagosome-lysosome fusion regulator.

To provide further support for the role of VAMP7 in autophagosome formation, given its additional roles in autophagosome maturation (Fader et al., 2009; Furuta et al., 2010), we studied the effects of VAMP7 knockdown in HeLa cells stably expressing an LC3-GFP-mRFP construct (mRFP and GFP are monomeric red fluorescent protein and green fluorescent protein, respectively) (Kimura et al., 2007; Sarkar et al., 2009). Because the two fluorescent proteins have different pKa values, this construct can be used as a probe for autophagic flux. At physiological pH, both proteins are stable, leading to both red and green fluorescence (autophagosome). On acidification, after fusion with the lysosomes, green fluorescence is rapidly lost because of the high pKa of GFP, and only red fluorescence remains (autolysosome) (Kimura et al., 2007; Sarkar et al., 2009). VAMP7 knockdown resulted in an increase in the numbers of autophagosomes (vesicles positive for both red and green) and a decrease in the numbers of autolysosomes (red positive, green negative) in the absence of Baf A1 (Figures 2C and 2D). This suggests a decreased delivery of autophagosomes to lysosomes due to VAMP7 knockdown, compatible with previous data (Fader et al., 2009; Furuta et al., 2010). However, there were fewer total LC3-positive vesicles in the VAMP7 knockdown cells, compatible with our earlier data suggesting that VAMP7 also regulates autophagosome formation. Indeed, we confirmed with this assay that VAMP7 knockdown impaired autophagosome formation by using Baf A1, where we saw a decrease in the number of autophagosomes (Figures 2C and 2D). It is worth noting that the number of autophagic vesicles per cell did not obviously increase in Baf A1-treated cells compared to untreated cells (Figure 2D). This result is due to homotypic fusion of mature autophagosomes, as described previously (Jahreiss et al., 2008). Indeed, the size of the autophagic vesicles increased under Baf A1 treatment, compared to untreated cells, and this correlates with a defect in autophagosome degradation (Figure S2E). The effects of VAMP7 knockdown on autophagosome formation (LC3-II levels under Baf A1 conditions) were also obvious under conditions of autophagy induction by starvation (Figure S2F), in which the knockdown decreased new autophagosome formation by about 40%.

We confirmed that the effects of VAMP7, syntaxin 7, and syntaxin 8 on autophagosome formation were not a consequence of impaired endocytosis (Figure S2G) (Ravikumar et al., 2010). However, Vti1b knockdown did slightly decrease endocytosis, which may confound interpretations of the exact functions of Vti1b (Figure S2G). p62/SQSTM1 is a protein involved in the formation of ubiquitin-positive cytoplasmic inclusion bodies and is constitutively degraded by the autophagic machinery. Therefore, the steady-state levels of p62 reflect the rate of autophagic degradation. Higher levels of p62 accumulated in VAMP7 and syntaxin 7 knockdown cells than in control cells (Figure S2H), indicating a functional role of VAMP7 and syntaxin 7 in autophagic activity. However, it is important to note that assays of autophagic substrate accumulation cannot discriminate between different sites of autophagic defects.

In order to understand the mechanism by which VAMP7 and associated SNAREs regulate autophagosome formation, we counted the number of endogenous Atg16L1 vesicles and studied the colocalization between Atg16L1 and LC3. Knockdown of VAMP7, syntaxin 7, syntaxin 8, and Vti1b increased the number of Atg16L1 vesicles per cell but decreased the colocalization between Atg16L1 and LC3 (Figure 2E and Figure S2I), in contrast to Ulk1 knockdown, a known inhibitor of Atg16L1 vesicle formation (Figure 2E) (Hara et al., 2008). The increased number of Atg16L1 vesicles observed after VAMP7, syntaxin 7, syntaxin 8, or Vti1b knockdown was specific to these SNAREs, as the knockdown of Sec22b, a SNARE involved in ER-Golgi vesicle trafficking (Hong, 2005), did not alter either the number of Atg16L1 vesicles per cell or the extent of Atg16L1/LC3 colocalization (Figure 2E). VAMP7 knockdown did not change the number of DFCP1 (double FYVE domain-containing protein 1) dots per cell, which are a marker of omegasomes, PI3P-positive structures originating from the endoplasmic reticulum, which are considered to represent a membrane/lipid source for autophagosomes additional to the plasma membrane (Figure S2J) (Axe et al., 2008). These data suggested that VAMP7, syntaxin 7, syntaxin 8, or Vti1b are involved in the maturation of Atg16L1 precursors toward phagophores.

### VAMP7 Regulates Homotypic Fusion of Atg16L1 Vesicles

Given the functional roles of VAMP7 and associated SNAREs on autophagic activity, and our observation that knockdown of these proteins increased the numbers of Atg16L1 vesicles, we hypothesized that these SNAREs regulate the maturation of Atg16L1 precursors by a mechanism that involves homotypic fusion events. To test this hypothesis, we performed live-cell imaging to study the homotypic fusion between Atg16L1 precursors.

**Figure 3:**
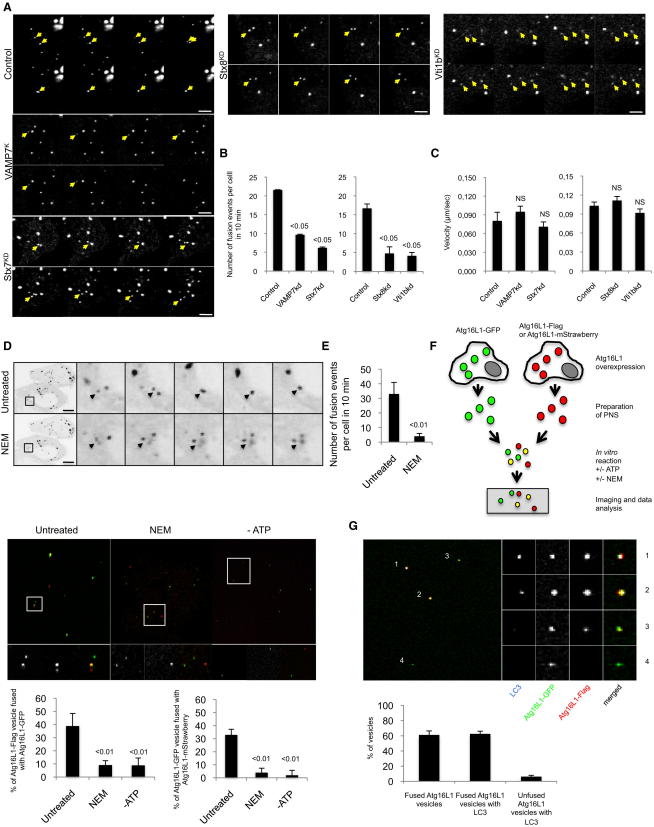
VAMP7 Mediates Homotypic Fusion of Atg16L1 Vesicle (A) HeLa cells transfected with control, VAMP7, syntaxin 7 (Stx7), syntaxin 8 (Stx8), or Vti1b siRNA for 4 days were transfected for 20 hr with Atg16L1-GFP. Representative images from 10 min movies are shown. Scale bars, 5 μm. (B) Quantification of the number of fusion events in 10 min (fusion and kiss and run) from the movies obtained in (A) is shown. Data represent the mean ± SEM of the number of fusion events per cell in 10 min. n = 5 cells for control, n = 6 cells for VAMP7 knockdown, n = 9 cells for Stx7 knockdown, n = 5 cells for Stx8 knockdown, and n = 6 cells for Vti1b knockdown. (C) The velocity of the Atg16L1-GFP vesicles from the experiment described in (A), obtained using ImageJ manual tracking plugin. Data represent the mean ± SEM of the velocity of 10 Atg16L1 vesicles. NS—not significant. (D) HeLa cells transfected with Atg16L1-GFP were subjected to live-cell imaging. Representative images (in inverted gray mode) from 10 min movies are shown (Untreated). Then, N-ethylmaleimide (NEM; 100 μM) was added to the medium and the acquisition started again for 10 min. Representative images from 10 min movies are shown (NEM). Arrows indicate fusion between two Atg16L1-GFP vesicles in untreated condition, whereas arrows indicate no fusion event in NEM-treated condition. Scale bars, 5 μm. (E) Numbers of fusion events in 10 min (fusion and kiss and run) from the movies obtained in (D). Data represent the mean ± SD of the number of fusion events per cell in 10 min. n = 5 cells for untreated cells, n = 5 cells for NEM-treated cells. (F) In vitro fusion assay of Atg16L1 vesicles. Postnuclear supernatants (PNS) of cells expressing Atg16L1-GFP were mixed with PNS from cells expressing either Atg16L1-Flag or Atg16L1-mStrawberry as indicated for 1 hr, in presence or absence of ATP and NEM. Representative confocal pictures are shown. The data represent the mean ± SD of the percentage of Atg16L1-Flag or Atg16L1-mStrawberry vesicles fused (colocalized) with Atg16L1-GFP. n = 202 vesicles for untreated condition, n = 170 vesicles for NEM-treated condition, n = 131 vesicles for ATP-untreated condition obtained from two independent experiments when the Atg16L1-Flag was used, n = 118 vesicles for untreated condition, n = 73 vesicles for NEM-treated condition, n = 31 vesicles for ATP-untreated condition obtained from 1 experiment when Atg16L1-mStrawberry was used. (G) After the in vitro fusion reaction of Atg16L1 vesicles, as described in (F), the vesicles were stained for endogenous LC3. A representative confocal picture is shown where two fused Atg16L1 vesicles (yellow dot) contain LC3 whereas one fused vesicle and one unfused vesicle (green) do not contain LC3. Graph represents the percentage of Atg16L1 vesicles containing LC3 depending on if they are fused (yellow) or unfused (green). Data are the mean ± SD. n = 66 vesicles obtained from two independent experiments. See also Figure S3 and Movie S1, Movie S2, Movie S3, Movie S4, Movie S5, Movie S6, Movie S7, and Movie S8.

**Figure S3:**
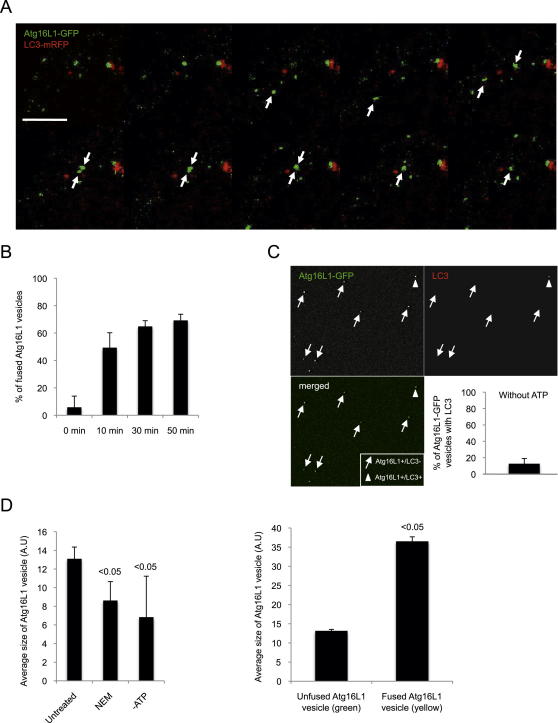
Homotypic Fusion of Atg16L1 Vesicles Analysis, Related to Figure 3 (A) HeLa cells transfected for 20 hr with Atg16L1-GFP and LC3-mRFP were subjected to live cell imaging. Representative images from 5 min movies are shown. Note that two Atg16L1-GFP vesicles without LC3 fused together. Arrows show Atg16L1-GFP vesicles involved in fusion event. Scale bar, 5 μm. (B) Time course of the in vitro fusion assay of Atg16L1 vesicles. Postnuclear supernatants (PNS) of cells expressing Atg16L1-GFP were mixed with PNS from cells expressing Atg16L1-Flag for 10 min, 30 min, or 50 min. The reaction were put on coverslips and analyzed by confocal microscopy after an immunostaining for Flag. The data represent the mean ± SD of the percentage of Atg16L1-GFP vesicles fused (colocalized) with Atg16L1-Flag. n = 30 vesicles for 0 min, n = 33 vesicles for 10 min, n = 79 vesicles for 30 min, n = 93 vesicles for 50 min obtained from two independent experiments. (C) Immunostaining of endogenous LC3 in the in vitro fusion assay of Atg16L1 vesicles without ATP. After the in vitro fusion reaction without ATP, the Atg16L1 vesicles were stained for endogenous LC3 and analyzed by confocal microscopy. A representative confocal picture is shown where Atg16L1 vesicles do not contain LC3 (arrows) whereas one Atg16L1 vesicle does contain LC3 (arrowhead). The graph represents the percentage of Atg16L1 vesicles with LC3. The data are the mean ± SD n = 60 vesicles obtained from two independent experiments. (D) Postnuclear supernatants (PNS) of cells expressing Atg16L1-GFP were mixed with PNS from cells expressing either Atg16L1-Flag for 1 hr, in absence of ATP or presence of NEM. The reaction was put on coverslips and analyzed by confocal microscopy. On the left, the data represent the average size ± SD of Atg16L1-GFP vesicles (arbitrary unit, A.U). n = 163 vesicles for untreated condition, n = 65 vesicles for NEM-treated condition, n = 159 vesicles for ATP-untreated condition obtained from two independent experiments. On the right, the data represent the average size ± SD of unfused Atg16L1-GFP vesicles with Atg16L1-Flag (only green) or fused Atg16L1-GFP vesicles with Atg16L1-Flag (yellow). n = 25 vesicles for unfused vesicles, n = 25 vesicles for fused vesicles. See also Movie S1.

We observed many fusion events between Atg16L1-GFP vesicles, which do not contain LC3 (Figure S3A and Movie S1), and a decrease in the fusion frequency in VAMP7, syntaxin 7, syntaxin 8, and Vti1b knockdown cells (Figures 3A and 3B and Movie S2, Movie S3, Movie S4, Movie S5, and Movie S6). The fusion defect observed in VAMP7, syntaxin 7, syntaxin 8, and Vti1b knockdown cells was not due to a defect in Atg16L1 vesicle movement, given that the velocity of the vesicles in VAMP7, syntaxin 7, syntaxin 8, and Vti1b knockdown cells was similar to control cells (Figure 3C). SNARE fusion is dependent on the action of αSNAP (an adaptor protein) and the AAA-ATPase NSF (*N*-ethylmaleimide-sensitive factor), which uses ATP hydrolysis to disassemble the SNARE complex (Martens and McMahon, 2008). As *N*-ethylmaleimide (NEM) treatment abolishes SNARE fusion, we treated Atg16L1-GFP-expressing cells with NEM and observed that the number of fusion events between Atg16L1-GFP vesicles was dramatically inhibited (Figures 3D and 3E and Movie S7 and Movie S8), compatible with a role of SNAREs in the Atg16L1 fusion process.

To confirm homotypic fusion between Atg16L1 precursors, we performed an in vitro fusion assay using postnuclear supernatants isolated from Atg16L1-GFP-expressing cells and separate lysates from either Atg16L1-Flag- or Atg16L1-mStrawberry-expressing cells. This assay relies on mixing of two fluorescently labeled organelles (green and red in our case), and fusion of the organelles results in mixing of the fluorescent probes resulting in double-labeled organelles (yellow). This assay cannot, however, detect fusion between identically colored (green-green or red-red) vesicles. Furthermore, multiple fusion events cannot be analyzed by this method. But the way this assay works is that the yellow vesicles are definitely fused. We observed time-dependent colocalization between Atg16L1-GFP vesicles and Atg16L1-Flag or Atg16L1-mStrawberry vesicles from 10 min to 60 min after the reaction started, indicating that homotypic fusion events occurred between the two populations of Atg16L1 vesicles in vitro (Figure 3F and Figure S3B). The presence of NEM or the absence of ATP in the in vitro fusion assay prevented the homotypic fusion of Atg16L1 vesicles, confirming a role of SNAREs in this fusion process (Figure 3F). Importantly, we observed that the definitely fused Atg16L1 vesicles (yellow) acquired LC3 much more efficiently than those that were red or green only, indicating the critical role of the homotypic fusion of Atg16L1 precursors in phagophore biogenesis (Figure 3G and Figure S3C).

Although fusion between identically colored Atg16L1 vesicles cannot be accurately analyzed by the method we have employed, we hypothesized that some fusions would occur between identically colored vesicles and that these vesicles should then be larger. We observed that the GFP-Atg16L1 vesicles in NEM-treated conditions or in ATP-depleted conditions (which both block fusion) are smaller than the GFP-Atg16L1 vesicles in the presence of ATP, suggesting that there were fusion events among green-alone and red-alone vesicles (Figure S3D). We also observed that the size of the yellow vesicles (definite fusion) was bigger than the size of the green vesicles alone (should have some self-fused and some unfused vesicles), confirming that Atg16L1 homotypic fusion increases the size of Atg16L1 vesicles (Figure S3D).

### VAMP7 Regulates the Size of Atg16L1 Vesicle in Cells

**Figure 4:**
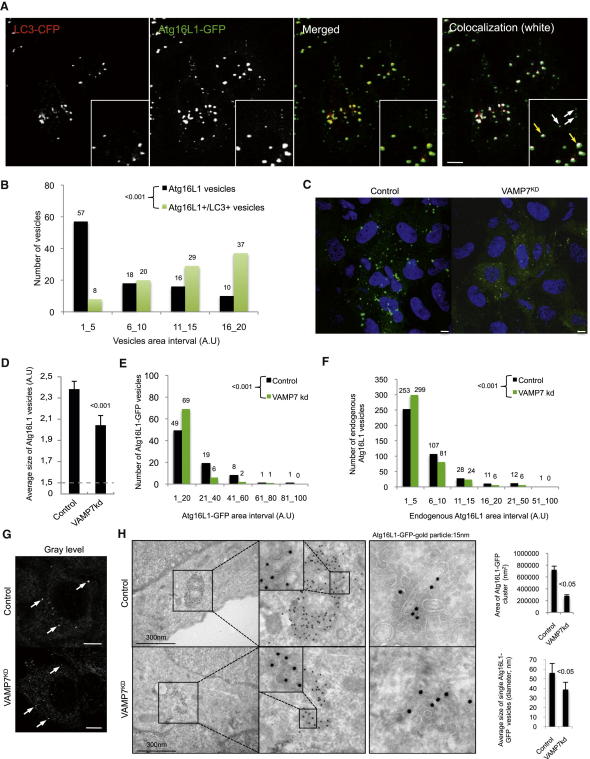
VAMP7 Regulates Atg16L1 Vesicle Size (A) HeLa cells were transfected for 20 hr with LC3-CFP (red) and Atg16L1-GFP (green). Confocal colocalization between LC3-CFP and Atg16L1-GFP is shown in white on the image. Yellow arrows show colocalization between LC3 and Atg16L1; white arrows indicate Atg16L1 vesicles with no LC3. Scale bar, 5 μm. (B) Size distributions of total Atg16L1 vesicles and Atg16L1^+^/LC3^+^ vesicles from images taken in (A) using ImageJ. n = 5 cells from two independent experiments. (C) HeLa cells transfected with control or VAMP7 siRNA for 4 days were transfected for 20 hr with Atg16L1-GFP. Representative confocal images are shown. Scale bar = 5 μm. (D) Atg16L1-GFP vesicle sizes from (C). Mean ± SEM of the average size of Atg16L1-GFP vesicles (A.U.: arbitrary unit); three independent experiments; at least 2000 cells were automatically analyzed. (E) Size distribution of Atg16L1-GFP vesicles from images taken in (C) using ImageJ. n = 4 cells for control and VAMP7 knockdown; two independent experiments. (F) HeLa cells transfected with control or VAMP7 siRNA for 5 days were starved for 4 hr. Size distribution of endogenous Atg16L1 vesicles is shown. n = 20 cells for control and VAMP7 knockdown; two independent experiments. (G) Representative images from the experiment described in (F). Arrows indicate endogenous Atg16L1 vesicles. Scale bar, 5 μm. (H) HeLa cells transfected with control or VAMP7 siRNA for 4 days were transfected for 20 hr with Atg16L1-GFP. The cells were processed for immunogold labeling with anti-GFP antibodies (15 nm gold particles) for electron microscopy. We observed Atg16L1-positive tubular structures in control cells, but not in VAMP7 knockdown cells (right-hand micrographs). Data represent mean ± SEM of the area of Atg16L1-GFP “cluster” (nm^2^) and the average diameter of individual Atg16L1-GFP vesicles (nm). n = 13 clusters for control. n = 19 clusters for VAMP7^KD^. n = 100 single vesicles for control. n = 100 single vesicles for VAMP7^KD^. See also Figure S4.

To investigate the relevance of the Atg16L1 precursor homotypic fusion in the maturation process toward a phagophore, we analyzed Atg16L1-GFP and LC3-CFP colocalization and examined the correlation between the size of Atg16L1 vesicles and the acquisition of LC3 in cells. We noticed that only the largest Atg16L1 vesicles acquired LC3 (Figures 4A and 4B), compatible with our in vitro data above (Figure S3D).

**Figure S4:**
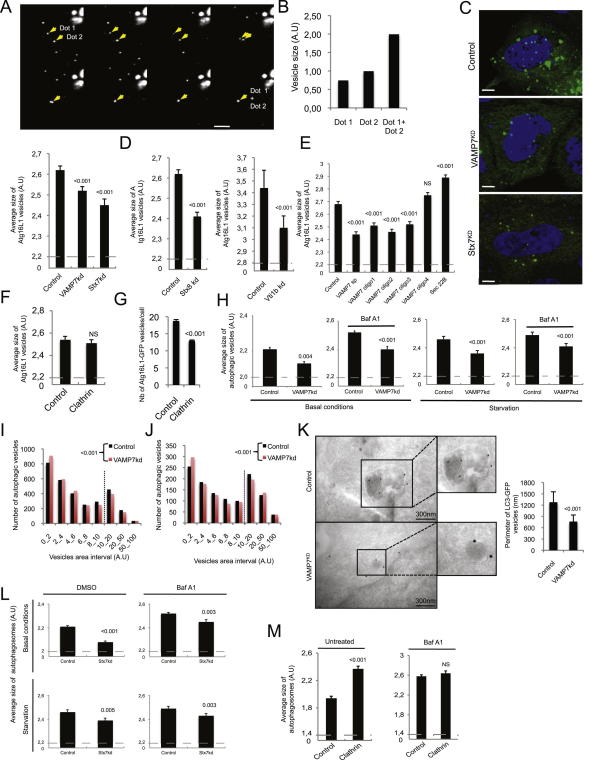
VAMP7, Syntaxin 7, Syntaxin 8, and Vti1b Regulate Atg16L1 and LC3 Vesicle Size, Related to Figure 4 (A) HeLa cells transfected with two rounds of control siRNA for 4 days were transfected for 20 hr with Atg16L1-GFP and subjected to live cell imaging. Representative images from 10 min movies are shown. Scale bar, 5 μm. (B) The graph represents the size of the vesicles, using ImageJ, identified as dot 1 and dot 2 in the images shown in top panel in (A). (C and D) HeLa cells transfected with two rounds of control, VAMP7, Syntaxin 7 (Stx7), syntaxin 8 (Stx8), or Vti1b siRNA for 4 days were transfected for 20 hr with Atg16L1-GFP. Cells were fixed and subjected to confocal analysis. Representative images are shown. Scale bar = 5 μm. Quantification of the size of Atg16L1-GFP vesicles is presented. The data represent the mean ± SEM of the average size of Atg16L1-GFP vesicles (A.U; abitrary unit) from three independent experiments (2 for Vti1b knockdown) where a minimum of 2000 cells were automatically analyzed using a Cellomics ArrayScan VTI HCS Reader. (E) HeLa cells transfected with two rounds of control, VAMP7 smartpool (sp), deconvoluted VAMP7 (Oligo 1 to Oligo 4) or Sec22B siRNA for 4 days were transfected for 20 hr with Atg16L1-GFP. Cells were fixed and subjected to a Cellomics ArrayScan VTI HCS Reader analysis. Quantification of the size of Atg16L1-GFP vesicles is presented. The data represent the mean ± SEM of the average size of Atg16L1-GFP vesicles (A.U) of one representative experiment from three independent experiments performed where a minimum of 2000 cells were automatically analyzed. (F) HeLa cells transfected with two rounds of control or clathrin siRNA for 4 days were transfected for 20 hr with Atg16L1-GFP. Cells were fixed and subjected to a Cellomics ArrayScan VTI HCS Reader analysis. Quantification of the size of Atg16L1-GFP vesicles is presented. The data represent the mean ± SEM of the average size of Atg16L1-GFP vesicles (A.U) from three independent experiments where minimums of 2000 cells were automatically analyzed. (G) Quantification of the number of Atg16L1-GFP vesicles/cell from the experiment described in (F) is presented. The data represent the mean ± SEM of the number of Atg16L1-GFP vesicles/cell (A.U) from three independent experiments where minimums of 2000 cells were automatically analyzed. (H) HeLa cells stably expressing LC3-GFP-mRFP were transfected with two rounds of control or VAMP7 siRNA for 5 days and either left untreated or starved for 4 hr. Cells were treated as indicated during the last 16 hr with bafilomycin A1 (Baf A1), fixed, and subjected to a Cellomics ArrayScan VTI HCS Reader analysis. Quantification of the size of autophagic vesicles is shown. The data represent the mean ± SEM of the size of autophagic vesicles (A.U; arbitrary unit) of one experiment representative of three independent experiments performed where minimums of 2000 cells were automatically analyzed. (I and J) Graph distribution of the size of autophagic vesicles from Baf A1-untreated cells from images taken in (H) using ImageJ. n = 20 cells for untreated condition and n = 10 cells for starvation condition. (K) HeLa cells stably expressing LC3-GFP-mRFP were transfected with two rounds of control or VAMP7 siRNA for 5 days and processed for immunogold labeling with anti-GFP antibodies (15 nm gold particles) for electron microscopy. Representative images are shown. Scale bar = 300 nm. The data represent the perimeter ± SD of LC3-GFP vesicles (nm). n = 13 vesicles for control. n = 19 vesicles for VAMP7^KD^. (L) HeLa cells stably expressing LC3-GFP-mRFP were transfected with two rounds of control or Syntaxin 7 (Stx7) siRNA for 5 days. Cells were treated as indicated during the last 16 hr with bafilomycin A1 (Baf A1), fixed, and subjected to a Cellomics ArrayScan VTI HCS Reader analysis. Cells were starved where indicated. Quantification of the size of autophagic vesicles is shown. The data represent the mean ± SEM of the size of autophagic vesicles (A.U) of one experiment representative of three independent experiments performed where minimums of 2000 cells were automatically analyzed. (M) HeLa cells stably expressing LC3-GFP-mRFP were transfected with two rounds of control or clathrin siRNA for 5 days. Cells were treated as indicated during the last 16 hr with bafilomycin A1 (Baf A1), fixed, and subjected to a Cellomics ArrayScan VTI HCS Reader analysis. Quantification of the size of autophagic vesicles is shown. The data represent the mean ± SEM of the size of autophagic vesicles (A.U; arbitrary unit) of one experiment representative of three independent experiments performed where minimums of 2000 cells were automatically analyzed. NS—not significant.

Because homotypic fusion results in the formation of larger vesicles in vitro (Figure S3D) and in cells (Figures S4A and S4B), we next used two different quantitative assays to study the sizes of Atg16L1 structures. The first assay used the HCS (high content analysis system) method, which allowed us to measure the sizes of more than 10,000 Atg16L1 vesicles per sample. We also assessed the Atg16L1 vesicle size distribution using ImageJ analysis of confocal pictures, which allowed us to work at a higher resolution. VAMP7, syntaxin 7, syntaxin 8, and Vti1b knockdown decreased the size of Atg16L1 vesicles (Figures 4C–4E, Figures S4C and S4D). We used deconvoluted siRNAs to confirm that the phenotype observed upon VAMP7 knockdown was not due to an off-target effect of the siRNA (Figure S4E), and we used an siRNA directed to the secretory SNARE Sec22B that did not reduce but increased the size of Atg16L1 vesicles to show that this was not a general consequence of SNARE knockdown (Figure S4E).

Importantly, we confirmed that VAMP7 knockdown decreased the size of endogenous Atg16L1 vesicles (Figures 4F and 4G). Finally, we performed immuno-electron microscopy (EM) analysis of Atg16L1 vesicles. The Atg16L1-immunogold labeling of normal cells revealed concentrations of staining that appear to label “clusters” of smaller vesicles (Figure 4H). However, these may also contain Atg16L1-positive tubulovesicular structures that are more connected, a phenomenon we have noted (Figure 4H; right pictures) The diameter of the individual small vesicles within the “clusters,” as well as the area of the entire “clusters,” are smaller in VAMP7 knockdown cells compared to control cells (Figure 4H), again suggesting that small Atg16L1 vesicles fuse together resulting in larger vesicles. The clustering of vesicles ensures close contact between the adjacent membranes, which, in analogous situations, is a prerequisite for the actual fusion process (Caplan et al., 2001). We, however, have also observed several individually distributed vesicles/tubules labeled by Atg16L1 in the control conditions (Figure 4H). Interestingly, the tubulovesicular Atg16L1 structures that we noted were completely absent in the VAMP7 knockdown cells, suggesting that prior fusion events are prerequisite for the elongation/maturation of the small Atg16L1 vesicles toward phagophores. Depending on what stage (clustering, docking, fusion, etc.) one captures with immuno-EM, it is possible to see individual vesicles or vesicles/tubules in clusters.

The effects of VAMP7, syntaxin 7, syntaxin 8, and Vti1b on Atg16L1 size are not simply due to unforeseen effects of these genes inhibiting endocytosis, as the size of the Atg16L1 vesicles in clathrin knockdown cells was normal, despite their numbers being reduced as we described previously (Figures S4F and S4G) (Ravikumar et al., 2010).

Next, to study whether the defect of Atg16L1 maturation observed in VAMP7 and syntaxin 7 knockdown cells had an impact on the size of mature autophagosomes, we used the HCS and ImageJ methods to measure the sizes of the autophagic vesicles (autophagosomes and autolysosomes) in HeLa cells stably expressing an mRFP-GFP-LC3 construct. Knockdown of VAMP7 and syntaxin 7 decreased the size of autophagic vesicles under basal conditions or starvation-induced conditions (Figures S4H–S4L). By contrast, clathrin knockdown (which is known to block autophagosome maturation, as well as affecting formation; Ravikumar et al., 2010) increased the size of the autophagic vacuoles, similar to what was observed when the cells were treated by Baf A1, as expected (Figure S4M).

**Figure 5:**
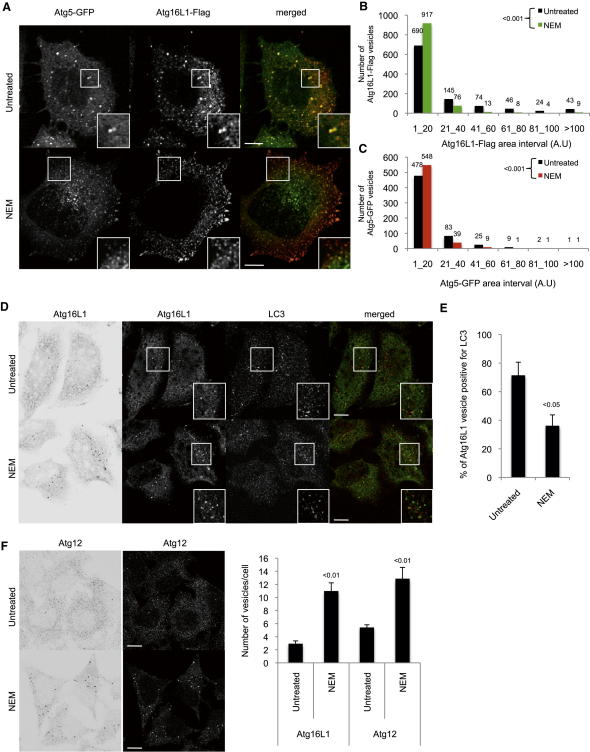
N-ethylmaleimide Treatment Decreases Atg16L1 Precursor Maturation (A) HeLa cells transiently transfected for 20 hr with Atg16L1-Flag and Atg5-GFP were treated as indicated with N-ethylmaleimide (NEM; 100 μM) for 10 min. Representative confocal pictures are shown. Scale bars, 5 μm. (B) Size distribution of Atg16L1-Flag vesicles from (A). n > 40 cells for untreated and NEM-treated cells; three independent experiments. (C) Size distribution of Atg5-GFP vesicles from (A). n > 20 cells for untreated and NEM-treated cells from three independent experiments. (D) HeLa cells were treated as indicated with N-ethylmaleimide (NEM; 100 μM) for 10 min. Cells were immunolabeled for endogenous Atg16L1 and LC3 and subjected to confocal analysis. Scale bars, 5 μm. (E) Colocalization efficiency between Atg16L1 and LC3 from (D). At least 50 cells were counted per experiment; data are the mean ± SD of the percentage of Atg16L1 vesicles containing LC3; three independent experiments. (F) HeLa cells were treated as indicated with N-ethylmaleimide (NEM; 100 μM) for 10 min. Cells were immunolabeled for endogenous Atg16L1 and Atg12 and subjected to confocal analysis. Scale bars, 5 μm. At least 50 cells were counted per experiment and the data are mean ± SD of the number of Atg16L1 or Atg12 vesicles per cell; three independent experiments. See also Figure S5.

**Figure S5:**
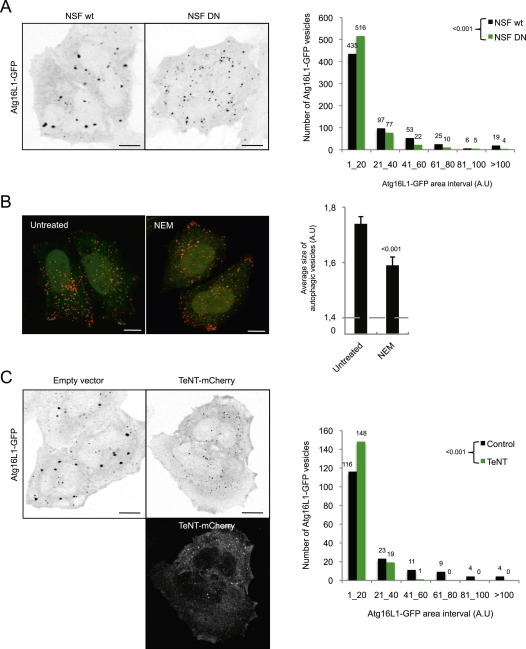
NEM and Tetanus Toxin Regulate Atg16L1 Vesicle Size, Related to Figure 5 (A) HeLa cells were transfected for 20 hr with Atg16L1-GFP and either wild-type NSF (NSF WT) or a dominant-negative mutant of NSF (NSF DN). Cells were fixed and subjected to confocal analysis. Representative images are shown. Scale bar = 5 μm. Graph distribution of the size of Atg16L1-GFP vesicles from confocal pictures using ImageJ is shown. n = 5 cells for NSF WT and NSF DN from two independent experiments. (B) HeLa cells stably expressing LC3-GFP-mRFP were treated with N-ethylmaleimide (NEM; 100 μM) for 10 min as indicated. Cells were fixed and subjected to a Cellomics ArrayScan VTI HCS Reader analysis. Representative confocal pictures and the quantification of the size of autophagic vesicles are shown. The data represent the mean ± SEM of the size of autophagic vesicles (A.U; arbitrary unit) of one experiment representative of three independent experiments performed where at least of 2000 cells were automatically analyzed. Scale bar, 5 μm. (C) HeLa cells were transfected for 20 hr with Atg16L1-GFP and either Tetanus neurotoxin-mCherry (TeNT-mCherry) or an empty vector. Cells were fixed and subjected to confocal analysis. Representative images are shown. Scale bar = 5 μm. Graph distribution of the size of Atg16L1-GFP vesicles from confocal pictures using ImageJ is shown. n = 3 cells for TeNT-mCherry and empty vector from two independent experiments.

Atg16L1-GFP, Atg16L1-Flag, and Atg5 vesicles were smaller in NEM-treated cells compared to untreated cells (Figures 5A–5C). Atg16L1 vesicles were also smaller in cells expressing a dominant-negative form of NSF factor (NSF DN), which blocks the fusion process (Skalski and Coppolino, 2005) (Figure S5A). Also, NEM-treated cells had more endogenous Atg16L1 and Atg12 vesicles but the Atg16L1-LC3 colocalization was decreased compared to untreated cells, compatible with a role of SNARE in Atg16L1 precursor maturation (Figures 5D– 5F). Similarly, NEM reduced the size of LC3-positive autophagosomes (Figure S5B).

To investigate whether other SNAREs were involved in the homotypic fusion of Atg16L1 precursors, we analyzed the effect of tetanus neurotoxin (TeNT) on the size of Atg16L1 vesicles. This toxin cleaves different SNARE proteins including VAMP1, VAMP2, and VAMP3 but not VAMP7 (Montecucco and Schiavo, 1993; Proux-Gillardeaux et al., 2005). In TeNT-expressing cells, the size of Atg16L1-GFP vesicles decreased compared to control cells (Figure S5C), suggesting that VAMP7 and its partner SNAREs may not be the only SNAREs involved in the homotypic fusion of Atg16L1 precursors.

### Hrb and the Longin Domain of VAMP7 Are Involved in Autophagosome Formation

**Figure 6:**
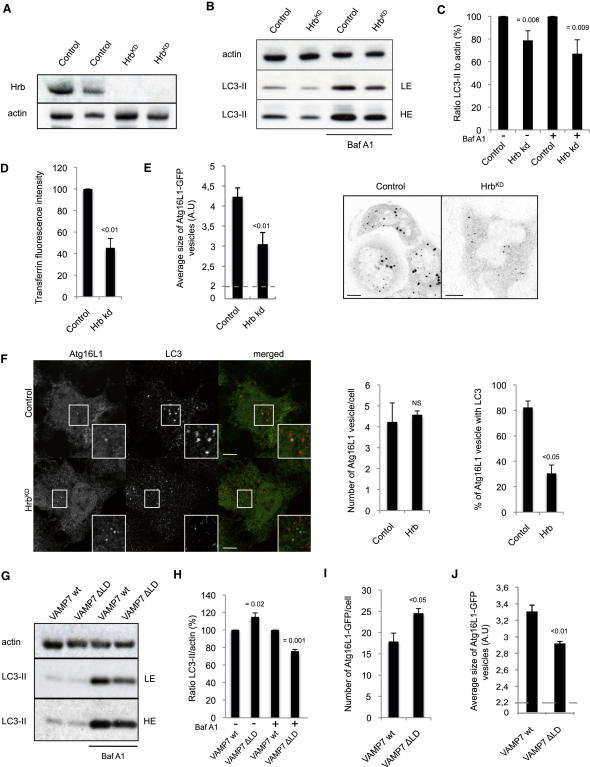
Hrb and the Longin Domain of VAMP7 Are Involved in Autophagosome Formation (A) HeLa cells transfected with control or Hrb siRNA for 5 days were lysed and subjected to western blotting with the indicated antibodies. (B) HeLa cells transfected with control or Hrb siRNA for 5 days were treated during the last 16 hr with bafilomycin A1 (Baf A1) or DMSO. Cells were lysed and subjected to western blotting with the indicated antibodies. (C) LC3-II/actin ratio from (B). Means ± SD of LC3-II/actin ratio; three independent experiments. (D) HeLa cells transfected with control or Hrb siRNA for 5 days were subjected to Alexa 488-labeled transferrin internalization assay; the data represent the mean ± SD of the transferrin fluorescence intensity; n = 2 independent experiments in triplicate. (E) Sizes of Atg16L1-GFP vesicles upon Hrb knockdown. The data represent the mean ± SEM of the average size of Atg16L1-GFP vesicles (A.U.: arbitrary unit); two independent experiments; at least 2000 cells were automatically analyzed. Representative confocal pictures are shown. Scale bars, 5 μm. (F) HeLa cells transfected with control or Hrb siRNA for 5 days were fixed and subjected to immunolabeling for endogenous LC3 (red) and Atg16L1 (green). Representative confocal pictures and Atg16L1 vesicle analyses are shown. At least 50 cells were counted per experiment; values are mean ± SD of the number of Atg16L1 vesicles per cell; two independent experiments. Colocalization efficiency between Atg16L1 and LC3. At least 50 cells were counted per experiment. Data are mean ± SD of the percentage of Atg16L1 vesicles containing LC3; two independent experiments. Scale bar, 5 μm. NS—not significant. (G) HeLa cells stably expressing VAMP7-HA (VAMP7 WT) or a truncated form of VAMP7-HA without the longin domain (VAMP7 ΔLD) were treated during 16 hr with bafilomycin A1 (Baf A1) or DMSO (vehicle). Cells were subjected to western blotting with the indicated antibodies. (H) Quantification of LC3-II/actin ratio from (G). Mean ± SD of the percentage of LC3-II/actin ratio; three independent experiments. (I) VAMP7 WT or VAMP7 ΔLD cells were transfected for 20 hr with Atg16L1-GFP. Cells were subjected to automated counting of Atg16L1 vesicles. Quantification of Atg16L1-GFP vesicles is shown. At least 2000 cells were counted per experiment and the values are mean ± SEM of one representative experiment of three independent experiments performed. (J) Sizes of Atg16L1-GFP vesicles in VAMP7 WT or VAMP7 ΔLD cells from (I). Data represent the mean ± SEM of the average size of Atg16L1-GFP vesicles (A.U.: arbitrary unit); three independent experiments; at least 2000 cells were automatically analyzed.

Given the role of VAMP7 and its partner SNAREs in Atg16L1 precursor homotypic fusion and maturation, we next considered where the pool of SNAREs came from. We previously demonstrated that Atg16L1 precursors could arise from the plasma membrane (Ravikumar et al., 2010). Therefore, we studied whether plasma membrane was a potential source of SNAREs for Atg16L1 precursor homotypic fusion. It has been previously shown that a pool of VAMP7, localized at the plasma membrane, is internalized via Hrb (Human immunodeficiency virus Rev-binding protein) (Chaineau et al., 2008; Pryor et al., 2008). VAMP7 interacts via its longin domain with Hrb, which in turn interacts with the clathrin adaptor AP-2 involved in clathrin-mediated endocytosis (Chaineau et al., 2008; Pryor et al., 2008). Knockdown of Hrb decreased the amount of LC3-II with or without Baf A1, indicating that Hrb regulates autophagosome formation (Figures 6A– 6C). In agreement with a previous report (Chaineau et al., 2008), knockdown of Hrb decreased transferrin internalization (Figure 6D). As shown for VAMP7, Hrb knockdown decreased the size of Atg16L1-GFP vesicles and the colocalization between Atg16L1 and LC3, without significantly affecting the number of Atg16L1 vesicles per cell (Figures 6E and 6F).

Autophagosome formation was decreased in a stable cell line expressing a truncated form of VAMP7-HA (VAMP7 ΔLD) without its longin domain (Gordon et al., 2009), compared to control cells expressing VAMP7-HA (VAMP7 wild-type [WT]) (Figures 6G and 6H). Moreover, the number of Atg16L1-GFP vesicles per cell increased, whereas their size was reduced in VAMP7 ΔLD cells, compared to VAMP7 WT cells (Figures 6I and 6J). These data indicated a role of the longin domain of VAMP7 in autophagosome formation, compatible with the Hrb effects above, and suggested that the plasma membrane represents one source of SNAREs involved in the homotypic fusion of Atg16L1 precursors.

### Autophagy Stimulation Induces Atg16L1 Vesicle Homotypic Fusion and Velocity

**Figure 7:**
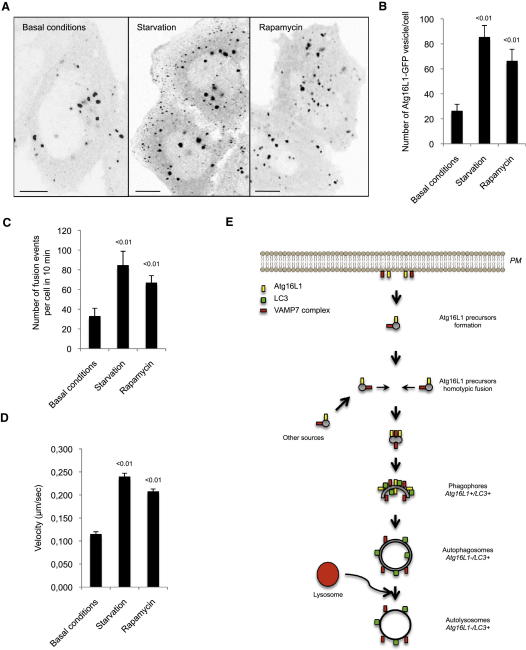
Autophagy Stimulation Induces Atg16L1 Vesicle Homotypic Fusion (A) HeLa cells transiently transfected for 20 hr with Atg16L1-GFP were treated as indicated with rapamycin (1 μg/ml) for 1 hr or starved for 1 hr. Representative confocal pictures are shown. Scale bars, 5 μm. (B) Quantification of the number of Atg16L1-GFP vesicles per cell using ImageJ from the pictures obtained in (A). Mean ± SD of the number of Atg16L1-GFP vesicle per cell; three independent experiments; n = 20 cells per experiment. (C) HeLa cells transiently transfected for 20 hr with Atg16L1-GFP were treated as indicated with rapamycin (1 μg/ml) for 1 hr or starved for 1 hr and subjected to live-cell imaging. Data are mean ± SD of the number of fusion events per cell in 10 min from two independent experiments. n = 5 cells for untreated condition, n = 7 cells for starvation condition, and n = 5 cells for rapamycin condition. (D) The velocity of the Atg16L1-GFP vesicles from (C) using ImageJ manual tracking plugin. Means ± SEM of the velocity of ten Atg16L1 vesicles. (E) Model of Atg16L1 precursor maturation. Atg16L1 precursors (Atg16L1^+^/LC3^−^) formed, in this example, from the plasma membrane undergo homotypic fusion via a VAMP7-containing SNAREs complex. Atg16L1 precursor homotypic fusion leads to LC3 acquisition and therefore maturation toward phagophores (Atg16L1^+^/LC3^+^) and fully formed autophagosomes (Atg16L1^−^/LC3^+^). See Movie S9 and Movie S10.

We showed that homotypic fusion of Atg16L1 precursor is required for autophagic precursor to mature toward a fully formed autophagosome. We next studied whether autophagy induction stimulated homotypic fusion and/or increased the velocity of the Atg16L1 precursors. We performed live-cell imaging of Atg16L1-GFP vesicles where the cells were starved or treated with rapamycin, a known autophagy inducer, in order to analyze the fusion events and the velocity of the vesicles. As previously described, starvation or rapamycin treatment increased the number of Atg16L1 vesicles per cell (Figures 7A and 7B) (Hosokawa et al., 2009). Starvation or rapamycin treatment increased the number of fusion events and the velocity of the Atg16L1 vesicles (Figures 7C and 7D and Movie S7, Movie S9, and Movie S10), suggesting a physiological role for SNAREs via the induction of the homotypic fusion of Atg16L1 precursors for autophagosome biogenesis.

## Discussion

In this study, we have identified a step in autophagosome biogenesis (Figure 7E). We have shown that Atg16L1 precursors (Atg16L1-positive and LC3-negative) undergo homotypic fusion. This increases the size of the vesicles, which appears to enhance LC3 acquisition and the maturation into autophagosomes. Atg16L1 precursor homotypic fusion is dependent on SNAREs, including VAMP7, syntaxin 7, syntaxin 8, and Vti1b. For Vti1b, one may need to be cautious when interpreting the data because knockdown of this protein has major effects on autophagosome-lysosome fusion (Furuta et al., 2010) and also affects endocytosis. However, VAMP7, syntaxin 7, and syntaxin 8 clearly regulated Atg16L1 vesicle fusion, Atg16L1 vesicle size, and autophagosome formation without affecting endocytosis. Although the knockdown of these SNAREs did not have very large effects on LC3-II formation, these were similar to what we saw when we knocked down Atg16L1. The effect of these knockdowns will be limited by the functional reserve allowed by the residual proteins (a phenomenon that is well described for SNAREs; Bethani et al., 2009), as well as the fact that even a complete abrogation of autophagosome formation in the presence of lysosomal inhibition will still allow an LC3-II signal from the autophagosomes present at the time the lysosomal inhibition commenced. It is likely that there are other SNAREs that participate in this step given that TeNT had an effect on the size of Atg16L1 precursor. This will be investigated in the future, as the key objective of the current study was to characterize the homotypic fusion of Atg16-positive autophagosome precursors and the importance of this process in autophagosome biogenesis.

These findings help to clarify how autophagosome membranes extend to eventually form vesicles that are much larger than the precursor structures from which they originate. Our data suggest that the membrane expansion can start to occur before the structures acquire LC3. However, this process does not necessarily preclude a requirement for membrane expansion on later LC3-positive structures. Indeed, LC3 can mediate membrane tethering and hemifusion, and these functions may be crucial for the expansion and/or assisting the final fusion of the double-membrane cups into fused vesicles (Nakatogawa et al., 2007).

Although previous studies have implicated a role for SNAREs in autophagy, these have been restricted to the autophagosome-lysosome fusion step (Fader et al., 2009; Furuta et al., 2010; Renna et al., 2011). Here we show a requirement for SNAREs during autophagosome biogenesis. It is worth pointing out that VAMP7 appears to play roles at both stages of the process. This scenario is similar to what we have recently found with components of the clathrin-mediated endocytosis machinery (Ravikumar et al., 2010). It is attractive to speculate that there are potential advantages for the same proteins to regulate autophagosome biogenesis and degradation, as this would help to coordinate autophagosome flux.

Our data with Hrb and with the VAMP7 longin domain mutant suggest that the plasma membrane is a potential source of the SNAREs required for the homotypic fusion of the autophagic precursor maturation. Indeed, VAMP7 could potentially be associated with Atg16L1 at the stage where it is still attached to the plasma membrane. It is possible that the transport of VAMP7 to the plasma membrane may regulate autophagy indirectly. For instance, VAMP7 can be found in the lysosomes and can be transported to the plasma membrane by exocytosis (Chaineau et al., 2009; Pocard et al., 2007). Also, direct transport of VAMP7 from the Golgi to the plasma membrane has also been described (Chaineau et al., 2009; Randhawa et al., 2004). However, we cannot exclude that a pool of VAMP7 from the secretory pathway fuses directly with the Atg16L1 precursors after their formation. All these possibilities will be interesting to study in the future.

Finally, we observed that autophagy induction by different stimuli triggered the homotypic fusion and the velocity of the Atg16L1 precursor. This observation suggests that a pool of autophagic precursors already present in the cell may be mobilized in response to autophagy-inducing stimuli.

In summary, our data suggest that the homotypic fusion of Atg16-positive, LC3-negative autophagosome precursors is a critical regulatory step in autophagosome biogenesis. This highlights the importance of membrane-trafficking events in autophagy prior to the formation of LC3, a step in the pathway that remains largely unexplored. It will be interesting to consider further how homotypic fusion of autophagosome precursors is regulated.

## Experimental Procedures

See the Extended Experimental Procedures for details of cell culture, antibodies and reagents, autophagy and endocytosis assays, microscopy, and statistics.

### Live-Cell Imaging

HeLa cells were seeded on 42 mm glass coverslips (PeCon, GmbH, Germany) at a density of approximately 1.5 × 10^5^ cells per coverslip. Cells were mounted in a POC chamber (PeCon GmbH) after which they were imaged immediately at 37°C. Imaging was performed on a Zeiss Axiovert 200 M microscope with a LSM 510 confocal attachment using a 63× 1.4 NA Plan Apochromat oil-immersion lens. Laser lines at 488 nm (Atg16L1-GFP) were used.

### In Vitro Fusion Assay of Atg16L1 Vesicles

The assay was performed as described with some modifications (Barysch et al., 2010). Briefly, two postnuclear supernatant (PNS) assays from HeLa cells expressing either Atg16L1-GFP and Atg16L1-Flag or Atg16L1-mStrawberry were mixed for 10 to 60 min in the presence of ATP and an ATP regenerative system, which can be visualized by immobilizing them on glass coverslips and subsequent confocal imaging in which double-labeled vesicles appear. The presence of ATP and NEM in the reaction allowed us to study the roles of ATP and SNAREs, respectively, in Atg16L1 vesicle homotypic fusion.

### Vesicle Size Assays

We used two methods to assess Atg16L1 or autophagic vesicle sizes. One used the Thermo Scientific Cellomics ArrayScan VTI HCS (high content analysis system) Reader and the Spot Detector Bioapplication protocol version 3, allowing analyses of >10,000 vesicles per sample, with a resolution of around 400 nm.

The second method used a confocal microscope. Vesicle sizes were quantified using ImageJ (Analyze Particles protocol), with a resolution of ∼150 nm. The data were statistically analyzed using Mann-Whitney test.

**Extended Experimental Procedures**

**Cell Culture**

HeLa cells were cultured in Dubelcco's modified Eagle's medium (DMEM) D6546 (Molecular Probes) containing 10% fetal bovine serum, supplemented with 2 mM L-glutamine and 100 U/ml Penicillin/Streptomycin in 5% CO_2_ at 37°C. HeLa cells stably expressing the LC3-GFP-mRFP protein and the VAMP7-HA protein were cultured in DMEM D6546 containing 10% fetal bovine serum supplemented with 2 mM L-glutamine, 100 U/ml Penicillin/Streptomycin and 500 μg/ml G418 (Sigma) in 5% CO_2_ at 37°C as previously described (Gordon et al., 2009; Kimura et al., 2007; Peden et al., 2004). HEK cells stably expressing the DFCP1-GFP protein were cultured as previously described (Axe et al., 2008).

**Antibodies and Reagents**

Antibodies include: rabbit anti-Atg16L1 (MBL International; PM040 and CosmoBio), mouse monoclonal anti-FLAG (Sigma; clone M2), mouse anti-GFP (BD Transduction Lab), rabbit anti-actin (Sigma), mouse monoclonal anti-tubulin (Sigma), rabbit anti-LC3 for western blot (Novus Biologicals- NB100-2220), mouse monoclonal anti-LC3 for immunofluorescence (MBL International; M152-3), rabbit anti-LC3 for immunofluorescence (Cell Signaling; #4108), mouse anti-p62 (BD Transduction Lab), goat anti-Hrb (Santa Cruz Biotechnology; sc-1424). Rabbit anti-VAMP7, rabbit anti-Syntaxin 7, rabbit anti-Syntaxin 8, rabbit anti-SNAP23, and mouse monoclonal anti-Vti1b have been previously described (Gordon et al., 2010).

Reagents include the following: bafilomycin A1 (Sigma), rapamycin (Sigma), N-ethylmaleimide (Sigma).

**Plasmids**

pFlag-Atg16L1, pEGFP-Atg16L1, pCFP-LC3, pmRFP-LC3, pmRFP-VAMP7, pGFP-Atg5, pmStrawberry-Atg16L1, pcDNA3.1 NSF, and pcDNA3.1 E329Q NSF have been described elsewhere (Cadwell et al., 2008; Kabeya et al., 2000; Mizushima et al., 1998; Skalski and Coppolino, 2005).

**Cell Transfection**

The cells were seeded at 1–2 × 10^5^ per well in 6-well plates and transfection was performed using LipofectAMINE (for DNA) or LipofectAMINE 2000 (for siRNA and double transfections with DNA and siRNA) (Invitrogen), using the manufacturer's protocol. For knockdown experiments, cells were transfected with siRNAs on day 1 and again on day 2 (two rounds of trasnfection). Predesigned siRNA were ordered from Thermo Scientific (Dharmacon Technologies) (siRNA IDs: VAMP7 - ON-TARGETplus SMARTpool and Set of 4, L-020864; Syntaxin 7 - ON-TARGETplus SMARTpool, LQ-019551; Syntaxin 8 - ON-TARGETplus SMARTpool, LQ-019873; Vti1b - ON-TARGETplus SMARTpool, L-020152; Hrb - ON-TARGETplus SMARTpool, L-011873; Sec22b - ON-TARGETplus SMARTpool, L-011963; Ulk1 - ON-TARGETplus SMARTpool, L-005049) or Applied Biosystems (siRNA IDs: clathrin heavy chain - 107565, s223262).

**Modulation of Autophagy**

To inhibit LC3-II degradation, cells were treated with bafilomycin A1 diluted in cell culture media to a working concentration of 400 nM for 4 hr or 200 nM for 16 hr, which is saturating for this effect (Sarkar et al., 2007). To induce autophagy in an mTOR-dependent manner, cells were amino acid- and serum-starved in Hanks balanced salt solution (HBSS; Sigma) for 1 to 4 hr or treated with rapamycin to a working concentration of 1 μg/ml for 1 hr.

**Western Blotting**

Cells were collected, rinsed with phosphate-buffered saline (PBS), and lysed on ice for 30 min in PBS containing 1% Triton X-100 and complete protease inhibitor cocktail (Roche). Lysates were centrifuged at 15,000 rpm for 5 min at 4°C, and supernatants were resolved by SDS-PAGE and transferred to PVDF membranes. The membranes were blocked with TBST (TBS 0.1% Tween-20) containing 1% nonfat dry milk and were then incubated overnight at room temperature with primary antibodies diluted TBST. Membranes were washed with TBST, incubated for 1 hr at room temperature with 2,500x dilutions of HRP-conjugated secondary antibodies (GE Healthcare Bioscience) in TBST containing 1% nonfat dry milk, and washed. Immunoreactive bands were then detected using ECL (GE Healthcare Bioscience).

**Fluorescence and Immunofluorescence Microscopy**

For immunofluorescence microscopy, cells were cultured on coverslips, fixed with 4% paraformaldehyde in PBS for 10 min or with ice-cold methanol for 10 min, and permeabilized with 0.1% Triton X-100 in PBS for 5 min. Coverslips were incubated with primary antibodies for 2 hr to 24 hr, washed three times with PBS, and incubated with secondary antibodies for 60 min. Samples were mounted using ProLong Gold antifade reagent with or without DAPI (Invitrogen) and observed using a Zeiss LSM510 laser confocal microscope. ImageJ (NIH) was used to count LC3 dots. BioImage XD was used for the colocalization analysis. Fifteen to twenty cells were analyzed for each experiment. Automatic counting of LC3 vesicles from HeLa cells stably expressing LC3-GFP-mRFP was performed using the Thermo Scientific Cellomics ArrayScan VTI HCS Reader and the Spot Detector Bioapplication protocole version 3.

**Endocytosis Assay**

Cells were collected and resuspended in ice-cold serum-free CO_2_-independent medium (SFM) containing 10 mg ml–1 BSA and centrifuged for 2 min at 1200 *g* and 4°C, after which they were resuspended in 300 μl of SFM/BSA containing Alexa-488 transferrin (Molecular Probes) and incubated on ice for 5 min for prebinding. They were then incubated for 5 min at 37°C to allow internalization. The cells were chilled on ice, centrifuged, and washed once with 700 μl SFM/BSA. The pellet was then resuspended in 300 μl of acid wash solution (0.1 M glycine, 150 mM NaCl, pH 3), incubated for 4 min on ice, centrifuged, and the process repeated. The pellet was then resuspended in chilled PBS/BSA and analyzed by fluorescence-activated cell sorting.

**Immunogold Electron Microscopy**

HeLa cells stably expressing VAMP7-HA (Gordon et al., 2009) were transfected with GFP-Atg16L1 for 24 hr. The cells were then fixed with a mixture of 2% paraformaldehyde and 0.2% glutaraldehyde in PBS for 2 hr, at room temperature. Cells were then prepared for ultrathin cryosectioning and immunogold-labeled, as previously described (Puri, 2009). Briefly, fixed cells were washed once in PBS/0.02 M glycine, after which cells were scraped in 12% gelatin in PBS and embedded in the same solution. The cell-gelatin was cut into 1mm blocks, infiltrated with 2.3 M sucrose at 4°C, mounted on aluminum pins and frozen in liquid nitrogen. Ultrathin cryosections were picked up in a mixture of 50% sucrose and 50% methyl cellulose and incubated with anti-HA and anti-GFP and revealed with 10 nm and 15 nm protein A gold (Utrecht).

**Statistical Analysis**

Significance levels for comparisons between groups were determined with t tests, repeated-measure, factorial ANOVA, or Mann-Whitney using the STATVIEW software, version 4.53 (Abacus Concepts, Berkeley, CA, USA).

## Supplemental Information

Movie S1. Live-Cell Imaging of Atg16L1 and LC3, Related to Figure 3 HeLa cells transfected for 20 hr with Atg16L1-GFP and LC3-mRFP were subjected to live-cell imaging. Scale bar, 5 μm. Time series: 30 frames/s for 5 min.

Movie S2. Live-Cell Imaging of Atg16L1, Related to Figure 3 HeLa cells transfected with two rounds of control siRNA for 4 days were transfected for 20 hr with Atg16L1-GFP. Cells were subjected to live-cell imaging. Scale bar, 5 μm. Time series: 30 frames/s for 10 min.

Movie S3. Live-Cell Imaging of Atg16L1 upon VAMP7 Knockdown, Related to Figure 3 HeLa cells transfected with two rounds of VAMP7 siRNA for 4 days were transfected for 20 hr with Atg16L1-GFP. Cells were subjected to live-cell imaging. Scale bar, 5 μm. Time series: 30 frames/s for 10 min.

Movie S4. Live-Cell Imaging of Atg16L1 upon Syntaxin 7 Knockdown, Related to Figure 3 HeLa cells transfected with two rounds of Syntaxin 7 siRNA for 4 days were transfected for 20 hr with Atg16L1-GFP. Cells were subjected to live-cell imaging. Scale bar, 5 μm. Time series: 30 frames/s for 10 min.

Movie S5. Live-Cell Imaging of Atg16L1 upon Syntaxin 8 Knockdown, Related to Figure 3 HeLa cells transfected with two rounds of syntaxin 8 siRNA for 4 days were transfected for 20 hr with Atg16L1-GFP. Cells were subjected to live-cell imaging. Scale bar, 5 μm. Time series: 30 frames/s for 10 min.

Movie S6. Live-Cell Imaging of Atg16L1 upon Vti1b Knockdown, Related to Figure 3 HeLa cells transfected with two rounds of syntaxin 8 siRNA for 4 days were transfected for 20 hr with Atg16L1-GFP. Cells were subjected to live-cell imaging. Scale bar, 5 μm. Time series: 30 frames/s for 10 min.

Movie S7. Live-Cell Imaging of Atg16L1, Related to Figure 3 HeLa cells transfected for 20 hr with Atg16L1-GFP were subjected to live-cell imaging. Scale bar, 5 μm. Time series: 30 frames/s for 5 min.

Movie S8. Live-Cell Imaging of Atg16L1 upon NEM Treatment, Related to Figure 3 HeLa cells transfected for 20 hr with Atg16L1-GFP were treated with N-ethylmaleimide (NEM; 100 μM) and subjected immediately to live-cell imaging. Scale bar, 5 μm. Time series: 30 frames/s for 10 min.

Movie S9. Live-Cell Imaging of Atg16L1 upon Starvation, Related to Figure 7 HeLa cells transfected for 20 hr with Atg16L1-GFP were starved for 1 hr and subjected to live-cell imaging. Scale bar, 5 μm. Time series: 30 frames/s for 5 min.

Movie S10. Live-Cell Imaging of Atg16L1 upon Rapamycin Treatment, Related to Figure 7 HeLa cells transfected for 20 hr with Atg16L1-GFP were treated for 1 hr with rapamycin (1 μg/ml) and subjected to live-cell imaging. Scale bar, 5 μm. Time series: 30 frames/s for 5 min.

Document S1. Article Plus Supplemental Information
